# The Effects of Calcium Nitrite on the Mechanical Properties and Microstructure of Early-Age Frozen Cement Paste

**DOI:** 10.3390/ma17102461

**Published:** 2024-05-20

**Authors:** Lijun Wan, Maopei Yu, Enze Wu, Yongqi Zhao

**Affiliations:** 1 Institute of Cold Regions Science and Engineering, Northeast Forestry University, Harbin 150040, China; yumaopei147@163.com (M.Y.); zhaoyongqi426@163.com (Y.Z.); 2 School of Civil Engineering and Transportation, Northeast Forestry University, Harbin 150040, China; 3 Heilongjiang Province Engineering Quality Road and Bridge Testing Center Co., Ltd., Harbin 150080, China; 15045869111@139.com

**Keywords:** cement paste, calcium nitrite, early freezing, compressive strength, microstructure

## Abstract

The objective of this paper is to investigate the effect of calcium nitrite (CN) on improving the mechanical properties and microstructures of early-frozen cement paste. Cement pastes containing 1%, 1.5%, 2%, 2.5%, and 3% CN were prepared. One batch of samples was frozen at −6 °C for 7 days and then cured at 20 °C, and the other batch of samples was directly cured at 20 °C as a control. The compressive strength, ultrasonic pulse velocity, and resistivity of all specimens at different target ages were measured under these two curing conditions. The hydration products and microstructures of typical samples were observed using XRD and scanning SEM. The results showed that the addition of 1.5% CN could promote cement hydration and enhance slurry densification, thereby increasing the compressive strength, ultrasonic pulse velocity, and electrical resistivity of the slurry, and positively affecting the early freezing resistance of the slurry. However, when the CN dosage exceeded 1.5%, the internal structure of the slurry was loose and porous due to the generation of a large amount of nitrite–AFm, which negatively affects the properties of the cement paste. In addition, the effectiveness of CN is only limited to temperature environments above −6 °C. Concrete antifreeze suitable for lower temperatures still requires further research.

## 1. Introduction

Cementitious materials have become the most widely used construction materials due to their high strength and excellent durability. However, in cold regions, early-age freezing damage has been one of the main causes of concrete performance deterioration [1]. When fresh cement paste is exposed to temperatures below −5 °C during the early stages, a significant amount of free water within the cement matrix freezes, leading to a near-complete halt in hydration reactions. Moreover, the freezing and subsequent expansion of the free water result in an increase in detrimental pore volume, causing severe microstructural deterioration [2]. Even after the paste thaws and hydration reactions resume, the ultimate strength is expected to be reduced by half [3,4]. Therefore, it is crucial to enhance the early frost resistance of freshly mixed concrete under cold weather conditions.

Currently, in cold weather conditions, the following methods are commonly employed to prevent freezing damage to freshly mixed concrete in the early stages: (1) heating the raw materials [5,6], (2) insulating or heating the freshly poured concrete [7,8], and (3) using antifreeze admixtures or accelerators [9,10]. Among these methods, heating or insulation measures present challenges such as high fuel or electricity consumption, increased risk of fire or electric shock, air pollution, and low construction efficiency [11]. Consequently, an increasing number of researchers have focused their studies on the use of antifreeze admixtures or accelerators to overcome the drawbacks associated with heating or insulation measures.

Commonly used antifreeze admixtures or accelerators can be classified into chloride-based (such as calcium chloride) and non-chloride-based (such as sodium bisulfate and calcium formate) types based on their chemical composition [12]. Calcium chloride has long been recognized as a highly effective accelerator for cementitious materials [13]. It has been widely used in cement concrete since 1886 and can significantly accelerate the setting and hardening of cement paste [14]. However, calcium chloride introduces excessive chloride ions, thereby increasing the risk of steel reinforcement corrosion in concrete structures, limiting its application in construction [13]. Therefore, researchers have started to consider the application of non-chloride-based antifreeze admixtures more extensively. Sodium thiocyanate (NaSCN) is one of the most commonly used non-chloride-based accelerators and was the first commercially available non-chloride-based accelerator for winter use in North America [15]. Wise et al. [16] reported that at −5 °C, concrete specimens containing 3% NaSCN achieved a compressive strength 3.1 times higher than that of plain cement paste after 28 days. Hoang et al. [17] found that at 5 °C, the addition of small amounts of NaSCN, diethanolamine (DEA), and glycerol (Gly) increased the compressive strength of fly ash cement mortar by 65% at 2 days and 15% at 28 days. Khan and G [18] pointed out that the combined use of sodium bisulfate and calcium nitrate can increase the compressive strength of concrete after 7 days of freezing at −10 °C followed by 21 days of water curing by 144.68%. However, sodium bisulfate has drawbacks such as high cost, high toxicity, and potential risks of alkali–aggregate reaction (AAR), which can negatively affect the durability of concrete structures [14]. Calcium formate (C_2_H_2_O_4_Ca) is also an effective non-chloride-based accelerator [19,20]. M. Heikal [21] found that calcium formate can accelerate the formation of C-S-H gel, leading to an 83.3% increase in the compressive strength of the paste after 28 days compared to ordinary cement paste. Hemalatha and Sasmal [22] reported that the addition of 0.5% calcium formate could compensate for the low early strength of fly ash cement composites. The hydration heat and XRD test results indicated that the hydration reaction mechanism is influenced by the fly ash content. Wang et al. [20] found that adding calcium formate to fly ash and slag blended cement paste results in the early formation of calcium aluminate silicate hydrate (CaAl_2_Si_2_O_8_⋅4H_2_O) and more ettringite (AFt), which helps improve early strength. However, some studies have shown that the addition of calcium formate can lead to the development of microcracks, increased porosity and pore size in the later stages of cementitious materials, and a reduction in the formation of internal Friedel’s salt (Ca_4_Al_2_(OH)_12_Cl_2_·4H_2_O), increasing the risk of chloride ion ingress into the paste [23].

Calcium nitrite (CN) antifreeze admixture can effectively mitigate the aforementioned issues. Compared to calcium chloride, sodium thiocyanate and calcium formate, CN both reduces the risk of chloride attack and avoids potential problems such as alkali-aggregate reaction reaction (AAR) and the degradation of the microstructure of the slurry at a later stage. It is a good non-chlorinated antifreeze. CN, as a corrosion inhibitor, has been widely used in reinforced concrete [24]. Additionally, calcium nitrite also exhibits early strength and antifreeze properties [25]. De Schutter and Luo [26] reported that concrete containing a commercial corrosion inhibitor with calcium nitrite showed a 10% increase in compressive strength at 3 days compared to ordinary concrete, but they did not analyze the early strength mechanism of CN. Choi et al. [27] studied the mechanical properties of cement mortar with a high dosage of calcium nitrite and calcium nitrate (exceeding 7% of cement mass) at 10 °C and found a positive correlation between the compressive strength at 1 day and the CN dosage, although all CN mortars exhibited lower compressive strength at 14 days compared to ordinary mortar. Kim et al. [28] also reported that at 10 °C, the addition of 4% and 8% CN in mortar increased the compressive strength at 1 day by 36.8% and 100%, respectively, compared to ordinary samples. However, although the aforementioned studies investigated the influence of calcium nitrite on the strength development and hydration products of cementitious materials at low temperatures, the curing temperatures used were still above 0 °C. Li et al. [29] reported that concrete containing 7% CN was able to reach about 7.5 times the compressive strength of normal concrete after 7 d of freezing at −5 °C. Yasien et al. [30] reported that concrete containing a mixture of silica nanoparticles, calcium nitrite, and calcium nitrate antifreeze could achieve a compressive strength of 38.9 MPa when cured at −5 °C for 28 d. Although the above two articles have demonstrated that CN can enhance the frost resistance of concrete in negative temperature environments, there are still fewer studies on the effect of CN on early-frozen cement paste. In conclusion, further research is needed to study the enhancement effect and mechanism of calcium nitrite on the early frost resistance of cementitious materials.

Therefore, the main objective of this study is to investigate the improvement effect and mechanism of calcium nitrite on early-frozen cement paste from both mechanical performance and microstructure perspectives. The research methods include setting time, compressive strength, ultrasonic pulse velocity, electrical resistivity, X-ray diffraction, and scanning electron microscopy. The innovations of this paper are as follows: (1) the investigation of the influence of calcium nitrite on the strength of cement paste under early freezing conditions at −6 °C, (2) the revelation of the impact of calcium nitrite on the hydration products and microstructure of early-frozen cement paste through microscopic experiments, and (3) the conclusion drawn from comprehensive experimental data that the optimal dosage of calcium nitrite under −6 °C early freezing conditions is 1.5%.

## 2. Materials and Methods

### 2.1. Raw Materials

**Figure 1:**
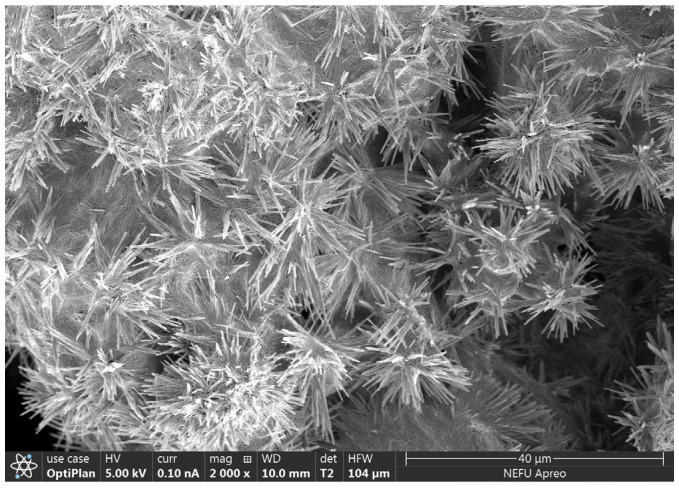
Microscopic morphology of calcium nitrite.

The experiment utilized P.O.52.5-grade ordinary Portland cement with a specific surface area of 377 m^2^/kg, and its chemical composition is presented in Table 1. Calcium nitrite was obtained from Beijing Jintong Letai Chemical Products Co., Ltd. (Beijing, China). Its particle size range was roughly around 0.15–0.28 mm, and its microstructure is depicted in Figure 1. In addition, in order to catch the w/b ratio and setting time of the cement paste by Chinese standard JTG/T 3650-2020 [31], a composite additive was also used to reduce the water and delay the setting time. The specific requirements of JTG/T 3650-2020 for the performance of cement paste are shown in Table 2. 

### 2.2. Paste Preparation and Curing

All of the specimens were prepared according to the mixing proportions specified in Table 3. The specimens were prepared according to the requirements of JTG/T 3650-2020 (the mixing speed ≥ 1000 r/min). Firstly, cement, compound additive, and calcium nitrite (at 0%, 1%, 1.5%, 2%, 2.5%, and 3% by mass of binders) were added to the mixer and stirred at low speed for 30 s. Subsequently, water was added to the mixer and stirred at high speed for 5 min at a speed of 1000 r/min. Finally, the fresh slurry was poured into a 40 mm × 40 mm × 160 mm mold. The specimens were named CN0, CN1, CN1.5, CN2, CN2.5, and CN3 according to the dosage of calcium nitrite. In order to investigate the improvement effect of CN on the early freezing resistance of cement paste, a batch of specimens was stored in a constant temperature refrigerator at −6 °C for 7 d immediately after specimen pouring and then placed in a standard curing chamber (20 ± 1 °C, 95% relative humidity) for 3 d, 7 d, and 28 d. For the convenience of the later discussion, the curing ages of the frozen cement paste were described as “−7 + 3 d”, ‘−7 + 7 d’, and ‘−7 + 28 d’, respectively. Another batch of samples was directly standard cured for 3 d, 7 d, and 28 d as a control.

### 2.3. Test Methods

**Figure 2:**
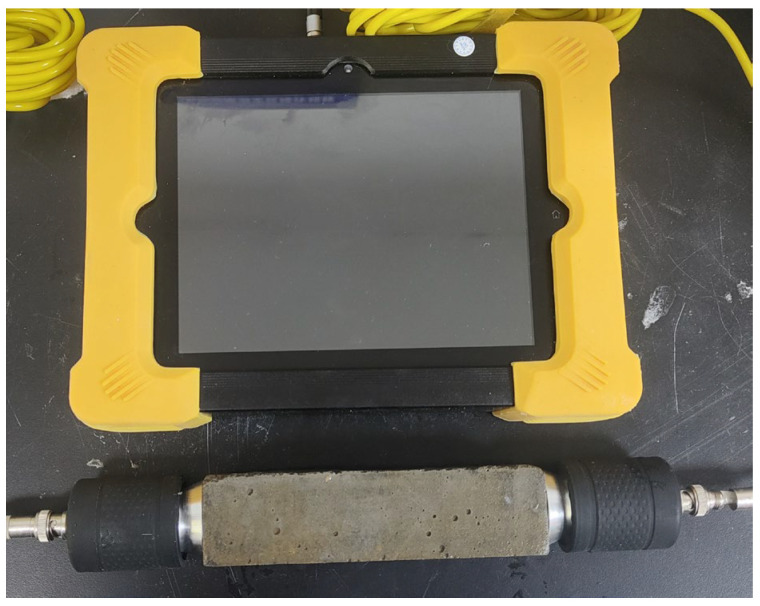
HC-U81 concrete crack defect tester.

**Figure 3:**
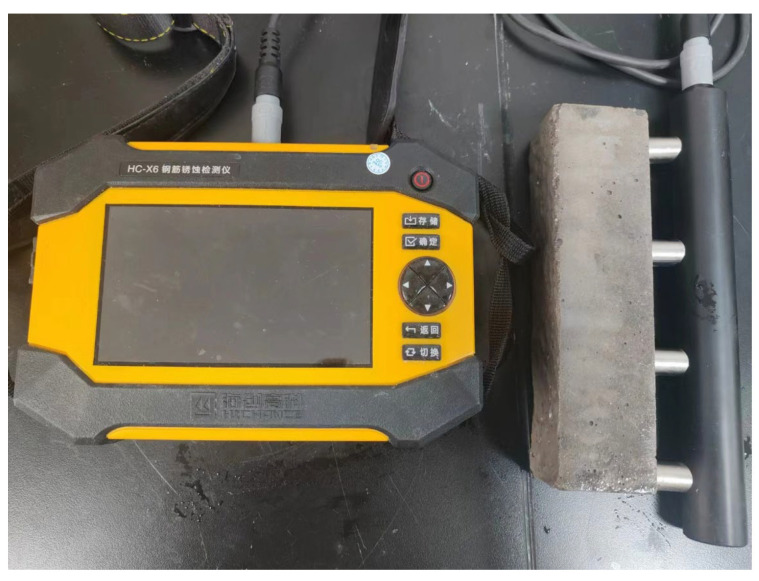
HC-X6 rebar corrosion detector.

According to the Chinese standard GB/T 1346-2011 [32], the initial and final setting times of cement paste were measured using a Vicat apparatus. The compressive strength test was conducted in accordance with the Chinese standard GB/T 17671-2021 [33]. Specimens reaching the specified curing age (40 mm × 40 mm × 160 mm) were cut into 40 mm × 40 mm × 40 mm specimens for the compressive strength test. The HC-U81 concrete crack defect tester (as shown in Figure 2) was employed to measure the ultrasonic pulse velocity (UPV) of specimens with ages of 28 d and −7 + 28 d, including CN0, CN1, CN1.5, CN2, CN2.5, and CN3. The instrument can automatically calculate the UPV value by measuring the distance between the probes on both sides of the specimen and the ultrasonic transmission time. The test results can qualitatively reflect the internal structural damage and densification of the specimen. Three experiments were conducted for each group of specimens, and the average of the three measurements was taken as the final result. The HC-X6 rebar corrosion detector (as shown in Figure 3) was utilized to measure the electrical resistivity of specimens with ages of 28 d and −7 + 28 d, including CN0, CN1, CN1.5, CN2, CN2.5, and CN3. Measurement of resistivity was automatically accomplished by placing four electrodes in contact with the surface of the specimen. Three experiments were conducted for each group of specimens, and the average of the three measurements was taken as the final result. Samples were taken from the center of CN0, CN1, CN1.5, CN2, CN2.5, and CN3 specimens with a curing age of 28 d and −7 + 28 d. Anhydrous ethanol was then used to prevent the samples from continuing to hydrate. All samples were then placed in a drying oven and dried at 40 °C for 48 h. A portion of the dried sample was ground to 75 μm and used for XRD tests. The crystalline phase of the sample powder was characterized using an X-ray diffractometer (XRD-6100, Shimadzu Corporation, Kyoto, Japan) in the range of 5–70°. A Cu target radiation source (40 kV, 30 mA) was employed, with a scanning speed of 8° per minute and a step size of 0.02°. A field emission scanning electron microscope (Apreo 2, Thermo Fisher Scientific, Waltham, MA, USA) was used to observe the microstructure of the −7 + 28 d of CN0, CN1, CN1.5 and CN3 samples. Prior to the test, the dried specimens were crushed into small flakes with a diameter of 3–6 mm and sprayed with gold on the surface.

## 3. Result

### 3.1. Setting Time

**Figure 4:**
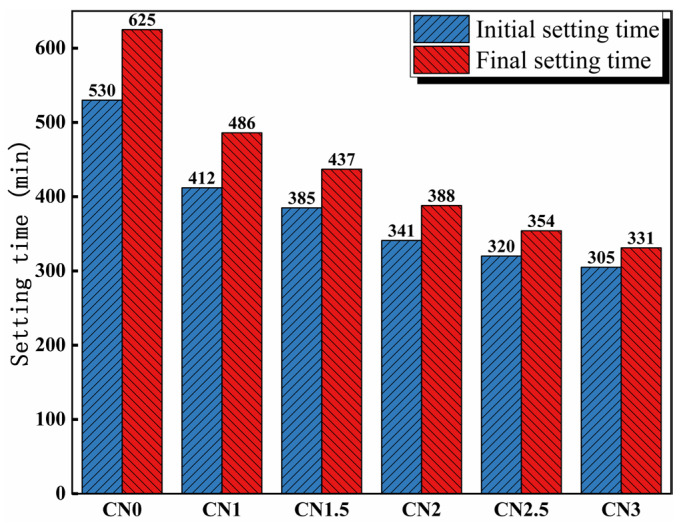
Influence of calcium nitrite content on setting time.

The influence of calcium nitrite on the setting time of cement paste is depicted in Figure 4. The results indicate that the addition of calcium nitrite significantly reduces the setting time of the cement paste. The initial setting time of the cement paste without the addition of calcium nitrite is 530 min, while the final setting time is 625 min. The initial setting times of the cement paste containing 1%, 1.5%, 2%, 2.5%, and 3% calcium nitrite are 412 min, 385 min, 341 min, 320 min, and 305 min, respectively. The corresponding final setting times are 486 mi, 437 min, 388 min, 354 min, and 331 min. This can be attributed to the acceleration of C_3_A hydration by NO_2_^−^, leading to the rapid formation of hydration products such as AFt and nitrite–AFm, which consume a significant amount of free water and accelerate the setting of the cement paste [34]. In addition, Ca^2+^ in CN has a nucleating effect [3]. Previous studies have shown that the nucleation effect can significantly promote cement hydration to accelerate slurry setting [35].

### 3.2. Compressive Strength

**Figure 5:**
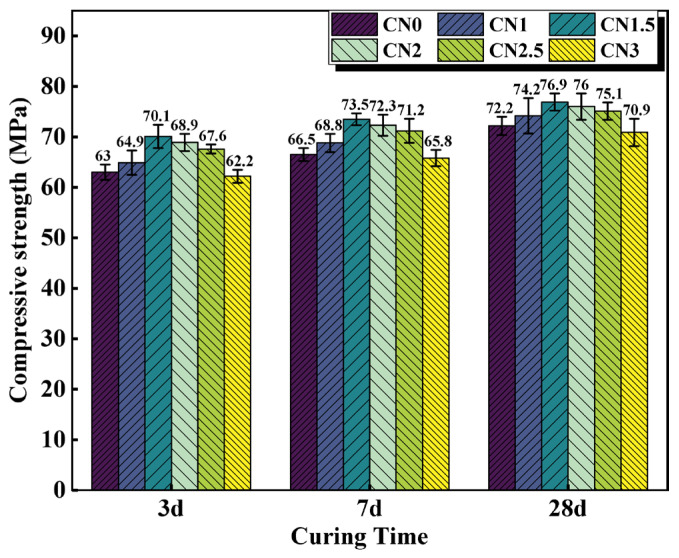
Compressive strength of CN cement paste cured at 20 °C for 3 d/7 d/28 d.

The compressive strength results of cement paste samples with different CN content are shown in Figure 5. The results indicate that under standard curing conditions, calcium nitrite slightly improves the compressive strength of cement paste at different ages. Moreover, with an increase in CN content, the compressive strength of the cement paste shows an initial increase followed by a decreasing trend. When the CN content is 1.5%, the compressive strength at 3 d, 7 d, and 28 d is the highest, measuring 70.1 MPa, 73.5 MPa, and 76.9 MPa, respectively. Compared to CN0, this represents an improvement of 11.3% (63 MPa), 9.4% (66.5 MPa), and 5.1% (72.2 MPa), respectively. However, when the content exceeds 1.5%, the beneficial effect of calcium nitrite on the compressive strength of the cement paste gradually diminishes. At a CN content of 3%, the compressive strength at each age even falls below that of CN0, reaching only 62.2 MPa, 65.8 MPa, and 70.9 MPa, respectively. This is because nitrite ions can accelerate the hydration of C_3_A, generated AFt, nitrite–AFm can make the cement matrix more dense by filling the pores, which has a positive effect on the development of the early strength of the specimen [27,34]. Meanwhile, CN contains the same Ca^2^+ as C_3_S and C_2_S, which can promote the hydration of C_3_S and C_2_S, leading to an increase in the production of C-S-H gel and Ca(OH)_2_, thus accelerating the rate of strength development [36]. However, when the dosage exceeds 1.5%, a large amount of CN can lead to an excessive formation of nitrite–AFm hydrates, exhibiting brittle fracture behavior and resulting in a decrease in strength [28]. In addition, by observing the microscopic morphology of CN in Figure 1, it can be found that CN has a fiber structure similar to that of ettringite [37]. Moderate amounts of ettringite can be used as a filler to reinforce the strength of the specimen, while excessive amounts of ettringite can cause the matrix to swell, resulting in a loss of strength [38,39]. Therefore, with the change in CN doping, its own mechanical effect may also lead to a change in specimen strength.

**Figure 6:**
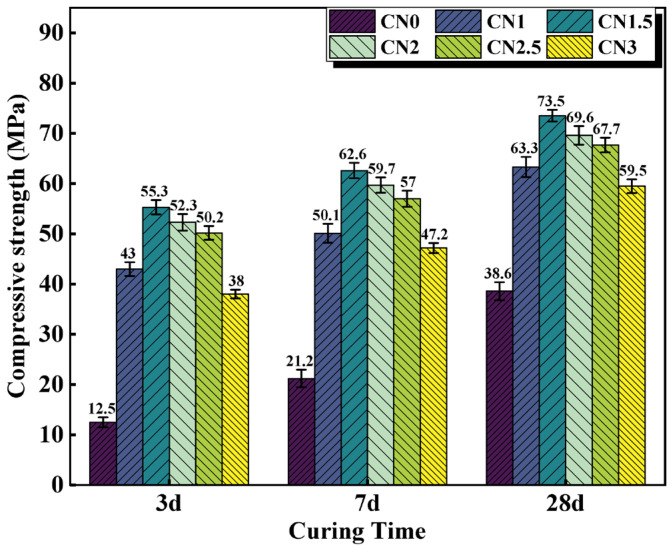
Compressive strength of CN cement paste cured at −6 °C for 7 d, then turned to 20 °C for 3 d/7 d/28 d.

Although the contribution of CN to the strength of standard cured cement paste is not significant, it is effective in improving the early frost resistance of cement paste. The compressive strength results of early-age frozen cement paste (−7 + 3 d/7 d/28 d) are shown in Figure 6. The results indicate that after early-age freezing damage, the compressive strength of ordinary cement paste at various ages significantly deteriorates. The compressive strength of the CN0 specimen at −7 + 3 d/7 d/28 d is 12.5 MPa, 21.2 MPa, and 38.6 MPa, respectively, which is only 19.7% (63.3 MPa), 31.7% (66.5 MPa), and 53.5% (72.2 MPa) of the compressive strength of CN0 at standard curing ages. This indicates that even if CN0 undergoes standard curing in the later stages, it cannot compensate for the damage caused by early low-temperature freezing to the cement paste. The addition of calcium nitrite can significantly improve the strength of early-age frozen cement paste at various ages. When the CN dosage is 1.5%, the compressive strength performs the best. The compressive strength of the CN1.5 specimen at −7 + 3 d/7 d/28 d reaches 4.4 times, 2.9 times, and 1.9 times that of CN0, respectively. Moreover, the compressive strength of the CN1.5 specimen at −7 + 28 d reaches 73.5 MPa, surpassing the compressive strength of the blank cement paste at 28 d under standard curing (72.2 MPa). It can be seen that the addition of CN almost offsets the impact of early freezing on the paste. This is because the addition of CN accelerates the formation of hydration products such as AFt, nitrite–AFm, C-S-H gel, and Ca(OH)_2_. The paste gains a certain strength before complete freezing occurs, and the freezing expansion of free water does not cause severe damage to the matrix structure. Upon thawing, cement hydration resumes, and the compressive strength continues to increase. Additionally, from the graph, it can be observed that when the CN dosage exceeds 1.5%, the specimens still experience a decrease in strength due to excessive formation of nitrite–AFm, but the overall strength remains significantly higher than that of CN0.

### 3.3. Ultrasonic Pulse Velocity

**Figure 7:**
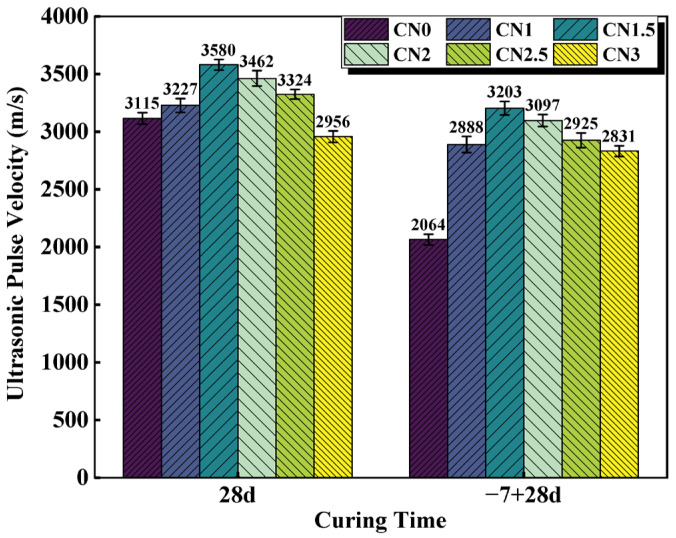
CN cement paste 28 d and −7 + 28 d UPV values.

UPV values vary with the porous structure of the material [40]. Higher UPV values indicate good material quality, while lower UPV values indicate that the structure may have many cracks or voids. [41]. The UPV results for the standard cured cement paste at 28 d and the early frozen cement paste at −7 + 28 d are shown in Figure 7. Wang and Wang et al. [42] claimed a positive correlation between UPV and compressive strength values, which is consistent with the results observed in this experiment. The experimental results show that the UPV of the standard-cured CN0 sample is 3115 m/s. As the CN content increases in the standard-cured CN cement paste, the UPV values gradually increase. When the CN content reaches 1.5%, the UPV value is maximized, reaching 3580 m/s. However, further increasing the CN content leads to a decrease in UPV values. The UPV of the CN3 sample decreases to 2956 m/s, which is 159 m/s lower than that of CN0. This is due to the fact that the cement hydration rate can be accelerated by an appropriate amount of CN. The produced hydration products, such as nitrite–AFm, C-S-H gel and Ca(OH)_2_, effectively reduced the number of internal pores, which resulted in a denser slurry structure and improved the UPV value. However, when CN doping exceeds 1.5%, the large amount of nitrite–AFm makes the structure loose, which makes the UPV value start to decrease.

Compared to the standard-cured 28 d cement paste, the UPV values of the frozen cement paste show varying degrees of decrease. Particularly for the CN0 specimen, its ultrasonic pulse velocity at −7 + 28 d is only 2064 m/s, which is a decrease of 1051 m/s compared to the standard-cured CN0. This indicates that early freezing at negative temperatures severely damages the microstructure of the cementitious system, leading to a decrease in the compactness of the paste. The presence of CN in the cement paste specimens significantly reduces the impact of early freezing. Among them, the UPV value of the early-frozen CN1.5 is 3203 m/s, even higher than that of the standard-cured CN0 (3115 m/s). However, it is worth noting that although the UPV values of the early-frozen specimens show a decreasing trend when the CN content exceeds 1.5%, the ultrasonic pulse velocity of the early-frozen CN3 sample is still significantly higher than that of early-frozen CN0. This is also mainly due to the fact that the addition of CN accelerates the generation of hydration products such as nitrite–AFm and improves the quality of the internal structure, thus avoiding damage to the slurry caused by free water freezing and expansion.

In summary, it is concluded that the UPV value of the CN1.5 specimen is the largest under both standard and early-freezing curing conditions. When the CN dosage exceeds 1.5%, the excessive generation of nitrite–AFm will, on the contrary, negatively affect the internal structure of the slurry, resulting in a decreasing trend of UPV values.

### 3.4. Electrical Resistivity

**Figure 8:**
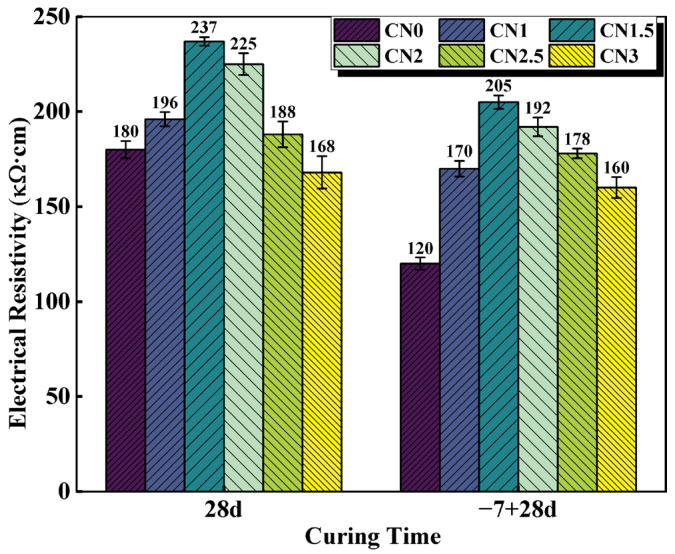
CN cement paste 28 d and −7 + 28 d electrical resistivity values.

The electrical resistivity of cementitious materials is largely dependent on the pore structure and increases with improved microstructural densification [43]. Therefore, resistivity measurements can provide insights into the microstructure of cementitious materials [44]. The resistivity results of the standard-cured cement paste at 28 d and the early-frozen cement paste at −7 + 28 d are shown in Figure 8. The trend of resistivity variation is similar to the observations from the previous UPV tests. Under standard-curing conditions, the resistivity of the CN0 specimen is 180 kΩ·cm. The CN1.5 paste exhibits the best hydration performance, with the most compact matrix structure and the highest resistivity, reaching 237 kΩ·cm. When the CN content exceeds 1.5%, the abundant generation of nitrite–AFm leads to a transition from a denser to a looser internal structure, resulting in a decrease in resistivity. The resistivity of the CN3 specimen is 168 kΩ·cm.

Under early freezing conditions, the resistivity of the CN0 specimen at −7 + 28 d shows a significant decrease, reaching only 120 kΩ·cm. The resistivity of the CN1.5 specimen at −7 + 28 d can increase to 205 kΩ·cm, which is a 70.8% improvement compared to the control group (CN0 specimen). As the CN content continues to increase, the resistivity starts to decrease but remains significantly higher than that of the standard early frozen cement paste. In addition, previous studies have shown that ionic conduction caused by water molecules may also lead to changes in specimen electrical resistivity [45]. Therefore, CN under early freezing conditions also contributes to increased specimen electrical resistivity by accelerating cement hydration and consuming more free water. It can be seen that the addition of CN can effectively improve paste densification and reduce the internal water molecule content, thus compensating for the decrease in cement paste electrical resistivity caused by early freezing.

### 3.5. XRD

**Figure 9:**
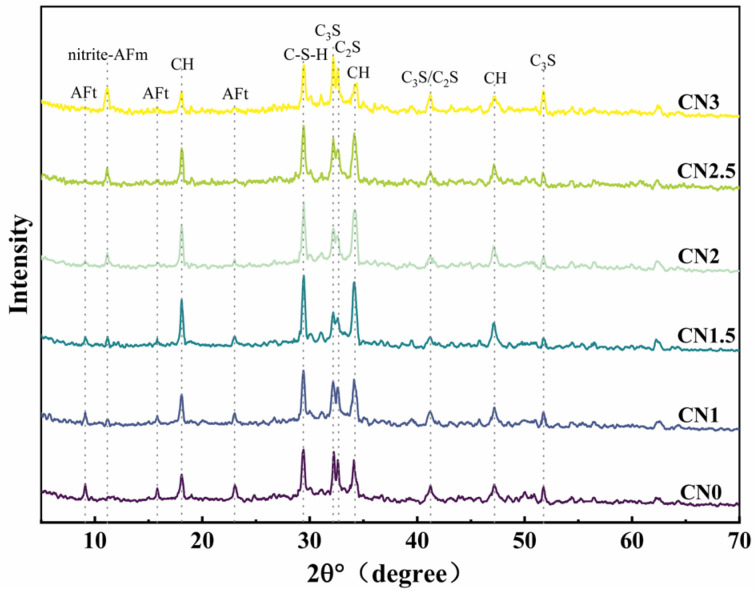
Standard-cured CN cement paste 28 d XRD pattern.

The XRD pattern of the CN cement paste at 28 days under standard curing conditions is shown in Figure 9. The main hydration products observed include ettringite, calcium hydroxide, C-S-H gel, and a new hydration product called nitrite–AFm [46,47]. From the graph, it can be observed that as the CN content increases, the content of ettringite (AFt) gradually decreases while the content of nitrite–AFm increases. When the CN content exceeds 1.5%, the presence of the AFt phase is almost negligible, and the nitrite–AFm phase starts to appear. When the CN content reaches 3%, the characteristic peaks of nitrite–AFm become more pronounced. This indicates that calcium nitrite enhances the hydration reaction rate of C_3_A and promotes the transformation of AFt to nitrite–AFm. Furthermore, with the increase in calcium nitrite content, the content of C-S-H gel and calcium hydroxide in each group initially increases and then decreases. When the CN content exceeds 1.5%, the content of C-S-H gel and calcium hydroxide starts to decrease and becomes lower than that of the control group when the CN content reaches 3%. This is because Ca^2+^ and NO_2_- can promote the hydration of C_3_A, C_3_S and C_2_S. However, when CN dosing exceeds 1.5%, the generation of a large amount of nitrite–AFm leads to the excessive consumption of free water in the cement paste, making the total amount of C-S-H gel with calcium hydroxide decrease. Meanwhile, the thermodynamically stable AFm will be wrapped around the surface of cement particles to reduce the dissolution and hydration space of C_3_S, which makes the hydration rate of C_3_S lower [48].

**Figure 10:**
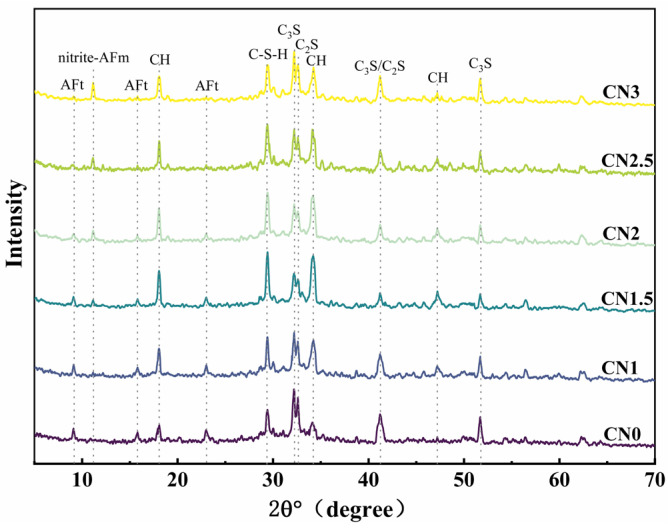
Early-age frozen CN cement paste −7 + 28 d XRD pattern.

The XRD pattern of the early-frozen CN cement paste at −7 + 28 d is shown in Figure 10. The results indicate that the hydration degree of the early-frozen cement paste containing CN is significantly better, and the variation trend of the hydration products’ content with increasing CN content is consistent with that of the standard-cured samples. It is worth noting that although the generation of C-S-H gel and calcium hydroxide still shows a decreasing trend when the CN content exceeds 1.5%, the hydration degree is always better than that of the control group. This is due to the fact that the water in the fresh slurry does not freeze immediately upon freezing. The cement paste containing CN had a faster rate of hydration and produced more hydration products than the control. After thawing, the hydration reaction continued in all specimens. Therefore, the cement paste containing CN had a higher content of hydration products at −7 + 28 d compared to the control. It can be seen that calcium nitrite promotes cement hydration more significantly at low temperatures compared to standard curing conditions.

### 3.6. SEM

**Figure 11:**
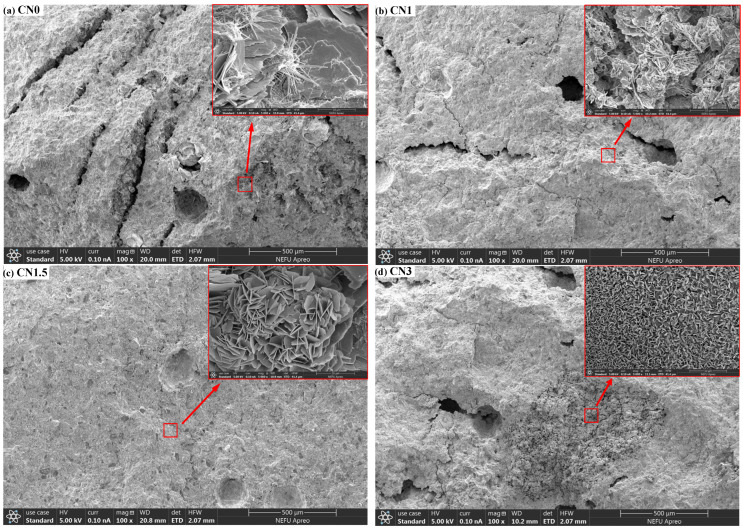
The microstructural morphology of CN0, CN1, CN1.5, and CN3 samples after −7 + 28 d of curing.

To investigate the influence of calcium nitrite on the microstructure of early-frozen cement paste, SEM observations were conducted on the micrographs of four groups of samples (CN0, CN1, CN1.5, and CN3) at −7 + 28 d, as shown in Figure 11. In the image of CN0 (Figure 11a), numerous long interconnected cracks and large pores can be observed on the sample surface. This is caused by the freeze–thaw expansion of free water in the fresh paste. When the surface of the sample is magnified, only a small amount of AFt can be observed in the cement paste. In comparison to CN0, CN1 (Figure 11b) shows a significant reduction in the number of cracks and pores, and needle-like ettringite is almost absent, replaced by some nitrite–AFm. This is consistent with the XRD results and indicates that CN accelerates the transformation of AFt to nitrite–AFm. The surface of CN1.5 (Figure 11c) is the most compact, with only a small number of small microcracks, and the quantity of nitrite–AFm is slightly increased compared to CN1. The number of cracks and pores on the surface of CN3 (Figure 11d) is between CN0 and CN1, but a significant portion of the sample surface appears extremely loose. When this part of the surface is magnified, a large amount of nitrite–AFm can be observed. This also confirms the previous discussion that an excessive amount of nitrite–AFm can lead to a porous and loose internal structure in the paste. The effect of CN dosing on the C-S-H content of the slurry was not observed in the SEM tests. Measurement of Ca/Si ratio in cement matrices using EDS can effectively assess changes in C-S-H content [49]. The degree of cement hydration will be further evaluated in future studies using EDS.

## 4. Discussion

From the above test results, it can be seen that the addition of CN will shorten the setting time. The appropriate amount of CN had a positive effect on the properties of both standard and early-freezing cured slurries. Moreover, the compressive strength, UPV value, and electrical resistivity of the slurry were maximized when the CN dosage was 1.5%. This can be attributed to the facilitating effect of CN on the hydration of C_3_A, C_3_S and C_2_S, which enhances the production of hydration products such as nitrite–AFm and C-S-H gels and is verified in the results of XRD and SEM microscopy experiments [50]. However, when CN doping exceeded 1.5%, it negatively affected the properties of the specimens, and the higher the CN doping, the more obvious the decrease in compressive strength, UPV value, and electrical resistivity of the slurry. This is because excessive CN doping causes the generation of a large amount of nitrite–AFm, which leads to a loose internal structure of the slurry [51]. This conclusion was also verified in XRD and SEM microscopic experiments. Therefore, the optimal dosing of CN is 1.5%.

To further evaluate the feasibility of calcium nitrite as an antifreeze. In this paper, CN is compared with conventional calcium chloride antifreeze in terms of efficiency, cost, and toxicity. (1) It has been shown that calcium chloride can increase the 28 d compressive strength of cement paste by about 13.3% to 44.8% at low temperatures [52]. However, the use of 1.5% CN in this paper increased the −7 + 28 d compressive strength of cement paste by 90.4%. (2) Normally, the recommended dosage of calcium chloride is 3% of the cementitious material at negative temperatures [53]. The price of commercially available calcium chloride is usually USD 277 per ton [54]. The results of this paper show that the optimal dosage of CN is 1.5% of the gelling material. The price of commercially available CN is USD 416 per ton. This means that for every ton of cementitious material consumed, the cost of using calcium nitrite is about the same as using calcium chloride. (3) Calcium chloride introduces chloride ions into concrete. However, the non-chlorinated antifreeze CN not only avoids the introduction of chloride ions but also effectively mitigates the rate of corrosion of reinforcing steel in concrete, extending the service life of the building. Therefore, it can be concluded that calcium nitrite is a more suitable antifreeze than calcium chloride in most cases. However, due to its toxicity, calcium nitrite cannot be used in engineering constructions related to drinking water and food [55]. 

## 5. Conclusions

In this paper, cement pastes with different CN dosages (0%, 1%, 1.5%, 2%, 2.5%, and 3%) were subjected to standard and early-freezing curing, respectively. Subsequently, the various properties of each group of specimens under the two curing conditions were compared with a view to assessing the improvement effect of calcium nitrite on the early freezing resistance of cement paste. The methods of study include setting time, compressive strength, ultrasonic pulse velocity, electrical resistivity, XRD and SEM. The main conclusions obtained are as follows:

(1) With the increase in CN content, the rate of cement hydration continues to accelerate, which leads to a gradual shortening of the cement paste setting time. The addition of an appropriate amount of CN can significantly improve the compressive strength of the slurry. Under standard curing conditions, the 28 d compressive strength of the CN1.5 specimens was 5.1% higher than that of ordinary cement paste. Under −6 °C early freezing conditions, the −7 + 28 d compressive strength of the CN1.5 specimens was 90.4% higher than that of ordinary cement paste. However, when the CN dosage exceeded 1.5%, the compressive strength of the specimens under both curing conditions began to show a decreasing trend.
(2) The results of the UPV test showed that the best structural densification of the slurry was achieved when the CN dosage was 1.5%. Under standard curing conditions, the 28 d UPV value of CN1.5 specimen was 14.9% higher compared with that of ordinary cement paste. Under the −6 °C early freezing condition, the −7 + 28 d UPV value of the CN1.5 specimen was 55.2% higher compared with that of ordinary cement paste.
(3) The electrical resistivity test also proved that the internal structure of the slurry was most dense when the CN dosage was 1.5. Under standard curing conditions, the 28 d resistivity of the CN1.5 specimen was 31.7% higher compared with that of ordinary cement paste. Under the early freezing condition at −6 °C, the electrical resistivity of the CN1.5 samples at −7 + 28 d was 70.8% higher than that of ordinary cement paste.
(4) The results of the XRD tests show that the appropriate amount of CN can promote the hydration of C_3_A, C_3_S and C_2_S, which increases the generation of hydration products such as nitrite–AFm, calcium hydroxide, and C-S-H gel. However, when the CN doping exceeded 1.5%, the generation of a large amount of nitrite–AFm would lead to a decrease in the total amount of C-S-H gel and calcium hydroxide.
(5) The results of the SEM tests show that many pores and cracks will appear inside the matrix after ordinary cement paste is subjected to early freezing, resulting in the deterioration of the microstructure. The addition of an appropriate amount of CN can promote the generation of nitrite–AFm and improve the early frost resistance of the slurry. The microstructure of the slurry is most dense when the CN doping is 1.5%. Further increasing the CN dosage will lead to the excessive generation of nitrite–AFm, which results in a loose and porous matrix structure.

Cement paste antifreeze for use in environments below −6 °C is under further study.

## Figures and Tables

**Table 1:** Chemical composition of cement (wt%).

Cement	CaO	SiO_2_	Al_2_O_3_	Fe_2_O_3_	MgO	SO_3_	K_2_O	Na_2_O	LOI
P.O.52.5	62.53	22.69	4.41	3.32	2.53	2.25	0.29	0.13	1.54

**Table 2:** JTG/T 3650-2020 performance requirements for cement paste.

Test Projects	Property Index
w/b ratio	0.26~0.28
Initial setting time (h)	≥5
Final setting time (h)	≤24

**Table 3:** Mix design.

Sample Name	Binders		CN (%) (Based on Binders Mass)	w/b Ratio	Water (g)
	PC (g)	Compound Additive (g)			
CN0	900	100	0	0.28	280
CN1			1		
CN1.5			1.5		
CN2			2		
CN2.5			2.5		
CN3			3		

## Data Availability

Data will be made available on request.
